# Validation of the Cardiosphere Method to Culture Cardiac Progenitor Cells from Myocardial Tissue

**DOI:** 10.1371/journal.pone.0007195

**Published:** 2009-09-25

**Authors:** Darryl R. Davis, Yiqiang Zhang, Rachel R. Smith, Ke Cheng, John Terrovitis, Konstantinos Malliaras, Tao-Sheng Li, Anthony White, Raj Makkar, Eduardo Marbán

**Affiliations:** Heart Institute, Cedars-Sinai Medical Center, Los Angeles, California, United States of America; Harvard Medical School, United States of America

## Abstract

**Background:**

At least four laboratories have shown that endogenous cardiac progenitor cells (CPCs) can be grown directly from adult heart tissue in primary culture, as cardiospheres or their progeny (cardiosphere-derived cells, CDCs). Indeed, CDCs are already being tested in a clinical trial for cardiac regeneration. Nevertheless, the validity of the cardiosphere strategy to generate CPCs has been called into question by reports based on variant methods. In those reports, cardiospheres are argued to be cardiomyogenic only because of retained cardiomyocytes, and stem cell activity has been proposed to reflect hematological contamination. We use a variety of approaches (including genetic lineage tracing) to show that neither artifact is applicable to cardiospheres and CDCs grown using established methods, and we further document the stem cell characteristics (namely, clonogenicity and multilineage potential) of CDCs.

**Methodology/Principal Findings:**

CPCs were expanded from human endomyocardial biopsies (n = 160), adult bi-transgenic MerCreMer-Z/EG mice (n = 6), adult C57BL/6 mice (n = 18), adult GFP^+^ C57BL/6 transgenic mice (n = 3), Yucatan mini pigs (n = 67), adult SCID beige mice (n = 8), and adult Wistar-Kyoto rats (n = 80). Cellular yield was enhanced by collagenase digestion and process standardization; yield was reduced in altered media and in specific animal strains. Heparinization/retrograde organ perfusion did not alter the ability to generate outgrowth from myocardial sample. The initial outgrowth from myocardial samples was enriched for sub-populations of CPCs (c-Kit^+^), endothelial cells (CD31^+^, CD34^+^), and mesenchymal cells (CD90^+^). Lineage tracing using MerCreMer-Z/EG transgenic mice revealed that the presence of cardiomyocytes in the cellular outgrowth is not required for the generation of CPCs. Rat CDCs are shown to be clonogenic, and cloned CDCs exhibit spontaneous multineage potential.

**Conclusions/Significance:**

This study demonstrates that direct culture and expansion of CPCs from myocardial tissue is simple, straightforward, and reproducible when appropriate techniques are used.

## Introduction

Congestive heart failure is a common clinical syndrome associated with significant morbidity, mortality and health care costs. Despite gains in other realms of cardiovascular disease, heart failure is the only cardiovascular disease that has been increasing in both incidence and prevalence [1]. The strategy of transplanting stem cells into damaged myocardium is emerging as a novel alternative in the treatment of this public health challenge. Ideal graft cells should be non-immunogenic, easy to expand *in vitro* and be able to engraft and differentiate into functional cardiac myocytes that couple electromechanically with the surrounding myocardium. Most importantly, transplanted graft cells should improve cardiac function and prevent ventricular remodelling. To date, a number of different cell types have been transplanted in experimental models, including fetal myocytes, embryonic stem cell derived myocytes, skeletal myoblasts, mesenchymal stem cells and several cell types derived from the bone marrow. Most recently, evidence that the human heart undergoes cardiomyocyte renewal throughout life has highlighted the potential of endogenous cardiac progenitor cells for cardiomyocyte replacement [2]–[4].

Our lab and others have been studying and refining methods to extract and expand CPCs directly from cardiac tissue [5]–[7]. Messina and colleagues first demonstrated that CPCs could be grown directly from cardiac tissue and further enriched within spherical aggregates, termed cardiospheres [8]. After injection into the injured heart, cardiospheres exhibit multilineage differentiation and confer functional benefits. Our group has advanced this method towards clinical translation by expanding cells from cardiospheres in monolayer culture, to yield cardiosphere-derived cells (CDCs) [5]. We have demonstrated that CDCs engraft, differentiate, secrete pro-angiogenic/cardiogenic cytokines and improve post-myocardial infarction (MI) function/perfusion [5], [6], [9]. This CDC technology has already entered phase 1 clinical trials (CADUCEUS; see ClinicalTrials.gov). Other groups have studied sub-populations within the initial outgrowth from cardiac samples by allowing cells to proliferate from the initial cardiac tissue and then selecting fractions for further study [10]–[12]. Cells from these sub-populations have been shown to be clonogenic and multipotent while remaining capable of self-renewal.

Several recent papers have varied the techniques used to proliferate, enrich and expand CPCs, with discrepant results [13]–. On the basis of these results, the validity of the cardiosphere method has been questioned. To clarify these important differences and to detail our experience with this culture technique, we outline the broad applicability of our CPC generation method, describe the effects of culture variations on phenotype, and use lineage tracing explore the origin of these promising therapeutic cells. We also demonstrate that CDCs are clonogenic, and that cloned CDCs are multipotent. Taken together, the findings support the validity of established cardiosphere-based methods for the generation of CPCs.

## Materials and Methods

### Ethics statement

This study was conducted according to the principles expressed in the Declaration of Helsinki. The study was approved by the Institutional Review Board of the Cedars-Sinai Medical Center and by the Institutional Review Board of the Johns Hopkins University. All patients provided written informed consent for the collection of samples and subsequent analysis. All animal procedures were conducted in accordance with humane animal care standards outlined in the NIH Guide for the Care and Use of Experimental Animals and were approved by the Cedars-Sinai Medical Center Animal Care and Use Committee and by the Johns Hopkins University Animal Care and Use Committee.

### Cell culture

Cardiac progenitor cells were cultured from human endomyocardial biopsies, adult bi-transgenic MerCreMer-Z/EG mice (12 weeks old; Jackson Laboratory, MA), adult C57BL/6 mice (10 weeks old; Harlan labs, Indianapolis, IN), adult GFP^+^ C57BL/6 transgenic mice (6–7 weeks old; Jackson Laboratory), Yucatan mini pigs (8 months old; S&S Farms, CA), adult SCID beige mice (8–9 weeks olds; Harlan Labs) and adult Wistar-Kyoto rats (8–9 weeks old; Harlan Labs), as previously described with several modifications [5], [8].

In the case of human percutaneous endomyocardial biopsy specimens, specimens were obtained during clinically-indicated procedures after informed consent under a protocol approved by the Institutional Review Board. Porcine endomyocardial specimens were sampled from the right ventricular septum using the bioptome in a similar manner. Biopsy specimens were stored on ice in high-potassium cardioplegic solution (5% glucose, 68.6 mM mannitol, 12.5 meq potassium chloride, 12.5 meq sodium bicarbonate, 10 units/mL heparin) to maintain tissue viability during transport. Tissue was processed within 2 hours.

In mice and rats, the hearts were excised with the animal under general anesthetic and the tissue was processed immediately. In a subset of WK rats, hearts were excised after heparinization (1000 U IV via inferior vena cava) and underwent retrograde perfusion with heparinized PBS to minimize thrombus formation prior to sectioning and washing with heparinized PBS.

The myocardial specimens were then cut into fragments less than 1 mm^3^, washed and partially digested with collagenase (MerCreMer-Z/EG mice, mini pigs, WK rats; 1 mg/ml; GIBCO, Carlsbad, Ca) or trypsin (human endomyocardial biopsies, C57 mice, GFP+ mice and SCID mice; 0.05%; GIBCO). These tissue fragments were culture as cardiac explants on fibronectin (20 µg/ml; Sigma) coated dishes in cardiac explant media (CEM; Iscove's Modified Dulbecco's Medium (GIBCO), fetal bovine serum (10% (mini swine only) or 20% (all other specimens); HyClone, Logan, UT), 100 U/ml penicillin G (GIBCO), 100 U/ml streptomycin (GIBCO), 2 mmol/l L-glutamine (Invitrogen, Carlsbad, CA), and 0.1 mmol/l 2-mercaptoethanol (GIBCO)). A subset of WK cardiac explants specimens were cultured in either 20% CEM or endothelial cell growth media (EGM2, Lonza, Walkerville, MD). After a variable period of growth, a layer of stromal-like cells emerged from the cardiac explant over which phase bright cells proliferated. The loosely-adherent cells surrounding the explant (termed cardiac outgrowth) were harvested using mild enzymatic digestion (0.05% trypsin under direct visualization or no more than 2 minutes, GIBCO). Cardiac outgrowth could be harvested up to four more times from the same specimen. For experiments using CDCs, cardiac outgrowth was seeded at 2×10^4^ cells/ml on poly-D-lysine coated dishes in cardiosphere growing media (CGM; 35% IMDM/65% DMEM-Ham's F-12 (GIBCO), 2% B27 (GIBCO), 0.1 mmol/L 2-mercaptoethanol(GIBCO), 10 ng/ml EGF(RD Systems), 20 ng/ml bFGF(PeproTech), 40 nmol/L Cardiotrophin-1 (RD Systems), 40 nmol/L thrombin (Sigma), 100 U/ml penicillin G, 100 U/ml streptomycin, 2 mmol/l L-glutamine). In all mini swine and a portion of the human endomyocardial biopsy specimens, cardiospheres were cultured in CEM with 10% FBS. Several days later, cells that remained adherent to the poly-D-lysine coated dishes were discarded, while detached cardiospheres were harvested, plated on fibronectin coated flasks and cultured in CEM (10% or 20%, as indicated above) to generate CDCs.

For single-cell cloning experiments, rat CDCs were first plated at an assumed density of 1 cell per well in a 96-well plate coated with fibronectin. Highly-proliferative clones were selected for subcloning, and subclones were plated at a visually-verified density of 1 cell per well. Clones were split (1∶5) when they reached confluency.

### Flow cytometry

Flow cytometry experiments were performed using benchtop flow cytometers (FACSCalibur and LSRII; BD Biosciences, San Jose, Ca). Monoclonal antibodies were labeled with fluorophores using commercial kits as required (Molecular Probes, Eugene, OR). The following monoclonal antibodies and conjugated fluorochromes were used with corresponding isotype controls: AA4 (AR32AA4, BD Pharmigen, San Jose, Ca), CD3 (MCA772FB, AbD Serotec, Oxford, UK), CD11b (MCA275FB, AbD Serotec), CD31 (sc-28188 Santa Cruz Biotech; BD Pharmingen 555445), CD34 (Chemicon CBL555F); CD45 (MCA340FB AbD Serotec; BD Pharmingen 555482), CD90-FITC (Dianova DIA120); c-Kit (sc-168 Santa Cruz Biotech; BD Pharmingen 550412), Ox-62 (MCA1029G, AbD Serotec). Fluorescent compensation was performed using single labeled controls. The percentage of positive cells was defined as the percent of the population falling above the 99th percentile of an isotype-matched antibody control cell population. All measures were performed using proprietary software (Flow-Jo 7.2.2 Treestar Inc., Ashland, OR, USA; CellQuest, BD Biosciences).

### Genetic fate mapping

Bi-transgenic MerCreMer-Z/EG mice were produced by crossbreeding cardiomyocyte-specific MerCreMer mice [16] and Z/EG mice [17] (Jackson Laboratory) [18]. Briefly, the Z/EG reporter mouse with cytomegalovirus (CMV) enhancer/chicken beta-actin promoter driving floxed beta-galactosidase followed by multiple stop codons and subsequently eGFP. Double heterozygous bi-transgenic MerCreMer-Z/EG mice were used for the myocyte lineage tracing experiments after induction of Cre recombination for GFP labeling exclusively in cardiomyocytes by 4-OH-Tamoxifen treatment [18]. Tamoxifen-treated bi-transgenic mice were used at the age of 10 week. Bi-transgenic hearts underwent the cell culture on 2-well chamber slides with immunostaining and microscopy performed at times indicated after plating. Animal genotype was verified by standard PCR on tail genomic DNA (primers available upon request).

### Immunostaining and microscopy

For immunohistochemistry, explants were cultured directly on fibronectin on 2-well chamber slides, fixed with 4% paraformaldehyde and stained in the whole mount. Cardiospheres were fixed in ethanol/acetone and stained in the whole mount. Confocal fluorescence imaging was performed on an Eclipse TE2000-U (Nikon, Melville, NY) equipped with a krypton/argon laser using UltraVIEW software (PerkinElmer, Boston, Mass). The following monoclonal antibodies were used: and GFP (sc-30200, Santa Cruz Biotech), cardiac troponin I (MAB3150,Chemicon), CD31 (550389, BD Pharmingen), CD34 (550390, BD Pharmingen), CD45 (555482, BD Pharmingen), CD90 (550402,BD Pharmingen), CD105 (MAB10971, R&D Systems), CD133 (CD133/1, Miltenyi Biotec), c-Kit (sc-168, Santa Cruz Biotech), connexin 43 (MAB3067, Chemicon), desmin (ab6322, Abcam), Ki67 (ab833, Abcam), MDR1 (MAB4334, Chemicon), myosin heavy chain (Rome, Italy10), Nkx2.5 (sc-14033, Santa Cruz), procollagen type I (MAB1912, Chemicon) and vimentin (AB1620, Chemicon). Secondary antibodies conjugated with Alexa fluorochromes were used.

### Statistical analysis

All data is presented as mean ± SEM. To determine if differences existed within groups, data was analyzed by a one-way ANOVA; if such differences existed, Bonferroni's corrected t-test was used to determine the group(s) with the difference(s) (G-B Stat software). A final value of P≤0.05 was considered significant for all analyses. All probability values reported are 2-sided.

## Results

### Cardiac progenitor cells can be reliably grown from myocardial tissue

We sectioned, enzymatically digested and plated myocardial tissue from human endomyocardial biopsies (n = 160), adult bi-transgenic MerCreMer-Z/EG mice (n = **6**), adult C57 mice (n = 18), adult GFP^+^ C57BL/6 transgenic mice (n = 3), Yucatan mini pigs (n = 67), adult SCID mice (n = 8) and adult Wistar-Kyoto rats (n = 80). Human ventricular biopsy specimens (21.0±15.9 mg) were obtained from patients who had undergone heart transplant (n = 59; 75% of recipients male (52±14 yo); 67% of donors male (32±12 yo)) or with unexplained cardiomyopathy (n = 12; 67% male; 49±15 yo).

**Figure 1:**
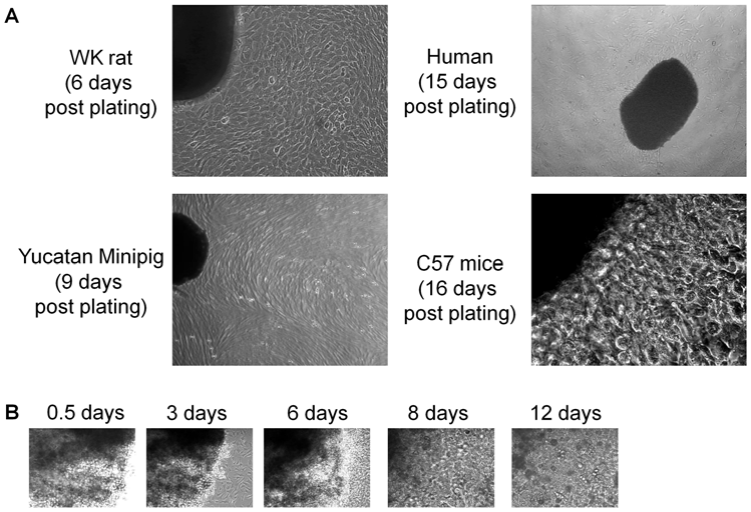
Culture cardiac explants. (A) Typical phase image examples of the spontaneous outgrowth from plated human, mouse, pig and rat tissue. (B) Serial imaging of MerCreMer-Z/EG mouse tissue cardiac explant tissue demonstrating the gradual rounding of margins and incorporation into the flattened outgrowth.

After several days in culture, a layer of stromal-like cells arose from adherent plated tissue (termed cardiac explants) over which small, round, phase-bright cells migrated. This surrounding layer of “outgrowth” cells became confluent within 7±2 days of plating mouse, pig and rat tissue, whereas human tissue typically became confluent 17±1 days after plating. As shown in Figure 1A, the gross histological appearance of the outgrowth from the plated tissue of different species was morphologically similar. Interestingly, we noted variable success proliferating outgrowth using specific strains of mice, particularly the C57BL/6 WT mice (Harlan) with typically only 20% successfully forming outgrowth. This difficulty was not noted when culturing tissue from transgenic mice (GFP^+^ C57BL/6 or MerCreMer-Z/EG) originally derived from the C57 strain.

### Cardiac progenitor cells are not of hematological origin

Given concerns that cardiac outgrowth may represent proliferation of retained hematological components [13], a subset of WK rats (n = 45) was heparinized prior to organ harvest. These hearts were then extracted and underwent retrograde perfusion with heparinized saline prior to sectioning and further washing. In all animals, we noted that outgrowth reliably grew in culture following organ perfusion and serial explant washing with heparin. This observation refutes the notion that outgrowth production is dependent upon the presence of retained circulatory elements.

**Figure 2:**
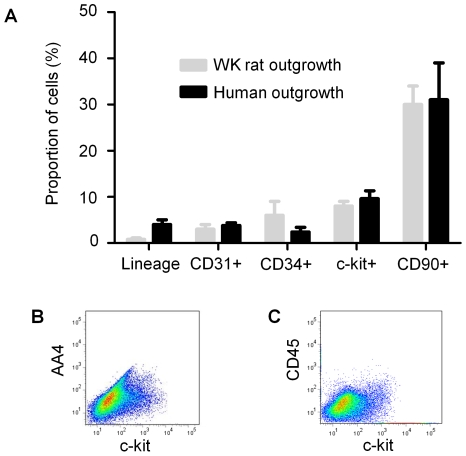
Flow cytometric analysis of the first outgrowth collection from cardiac tissue. (A) A comparison of the proportion of the initial human and WK rat outgrowth cells expressing markers of CPC (c-Kit), endothelial (CD31, CD34) and mesenchymal (CD90) origin. (B) Flow cytometry dot plot of the initial cellular outgrowth harvested from WK rat tissue demonstrating co-segregation of mast cell antigen AA4 with CPC marker c-Kit. (C) Flow cytometry dot plots of the initial outgrowth harvested from WK rat tissue demonstrating the co-segregation and abundance of c-Kit and CD45.

Once confluent, the outgrowth cells surrounding the explant were collected 3 times after the initial harvest using mild enzymatic digestion. As the plated explant tissues persisted in culture, they became progressively more rounded with less-defined margins (Figure 1B). Flow cytometry demonstrated that the initial outgrowth collected from human and rat tissue is enriched for sub-populations that antigenically resemble CPCs (c-Kit^+^), endothelial cells (CD31^+^, CD34^+^) and mesenchymal cells (CD90^+^). As shown in Figure 2A, the proportion of c-Kit^+^, CD31^+^, CD34^+^ and CD90^+^ cells falling above the 99th percentile of the control was not significantly different between human and rats. Importantly, these collections where negative for markers of hematological origin (hematological lineage screen), including AA4 (mast cell antigen; Figure 2B) and CD45 (leukocyte common antigen; Figure 2C). This finding indicates that hematological contamination does not constitute a major component of *ex-vivo* expanded CPCs.

### Variations in culture conditions alter culture efficiency and phenotype

To enhance the efficiency of CPC generation, we examined the influence of different culture conditions on culture output. While difficult to quantify, we have noted that mincing tissue more finely reliably enhances the generation of outgrowth and the eventual yield of collected cells. Initial digestion of the minced tissue also plays an important role in the amount of time required for outgrowth to attain confluence. The substitution of collagenase for trypsin significantly reduced the amount of time required for human cells to attain initial confluence (17.3±0.7 days vs. 22.1±1.5, respectively; p<0.01) and significantly increased outgrowth yields (7.7E+05±9.9E+04 vs. 8.7E+04±1.3E+04, respectively; p<0.01).

**Figure 3:**
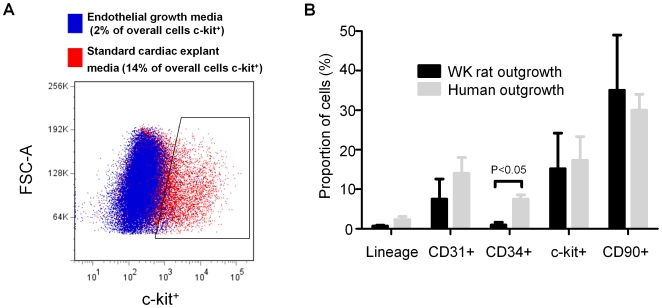
Effect of variation in culture methods on phenotype. (A) Effect of FBS content the proportion of the human CDCs expressing markers of CPC (c-Kit), endothelial (CD31, CD34) and mesenchymal (CD90) origin. (B) Flow cytometry dot plot demonstrating the influence of endothelial media and standard cardiac explant media on the proportion of WK rat outgrowth cells expressing the CPC marker c-Kit.

In contrast, alteration of the media from standard CEM to endothelial media markedly reduced both the yield (1.7E+06±1.1E+06 vs. 3.3E+05±3.1E+05; p<0.01) and the CPC content (c-Kit+ cells 15±7% vs. 3±1%, p<0.01) of the collected aggregate (Figure 3A). We noted that decreasing the FBS content of CEM from 20% to 10% reduced the proportion of CDCs expressing CD34 (7.5±1.2 vs. 0.9±0.7, respectively; p<0.01), but did not alter the other subpopulations within the human flow cytometry profile (Figure 3B).

### A portion of CPCs arise from myocardial de-differentiation

**Figure 4:**
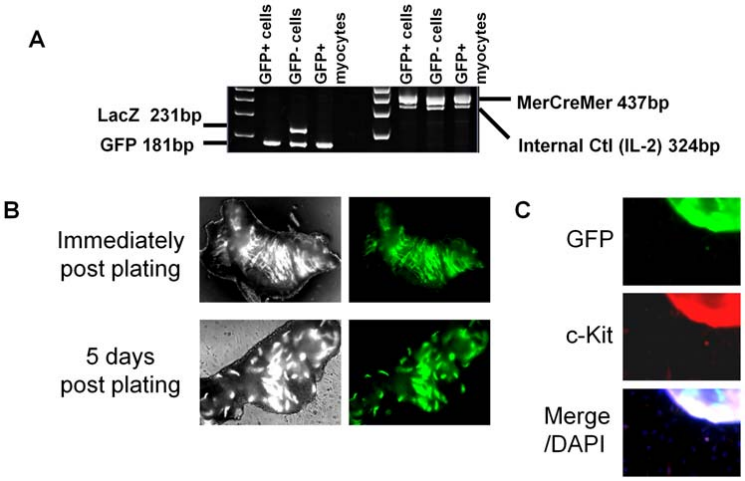
Lineage tracing of outgrowth from cardiac samples. (A) RT PCR depicting recombination following administration of tamoxifen. (B) Phase and fluorescent imaging of the outgrowth from MerCreMer-Z/EG mouse tissue 0 and 5 days after plating. (C) Immunohistochemistry depicting myocytes and non-myocyte derived c-kit^+^ cells after 2 weeks in culture. Inset lines highlight c-kit^+^ and GFP- cells.

To explore the ultimate origin of CPCs within outgrowth, we used a bi-transgenic MerCreMer-Z/EG mouse model with cardiomyocytes irreversibly GFP-labeled after Tamoxifen treatment (Figure 4A). As shown in Figure 4B and 4C, the outgrowth from plated tissue is established 5 days after plating with no detectable GFP fluorescence. Aggregate data were collected from >7 random fields to quantify the composition of cells in the initial outgrowth from myocardial samples. These data showed that 6.4±0.5% of the cells counted were c-kit^+^ but GFP^−^; such CPCs cannot have been of cardiomyocyte origin. Thus, cardiomyocytes are not present in the early outgrowth, and an appreciable fraction of the outgrowth cells are CPCs of non-cardiomyocyte origin. The contention that “contaminating myocardial tissue fragments” in the outgrowth are important is therefore rejected [14].

### Adult human cardiospheres exhibit a complex mixed cell phenotype

**Figure 5:**
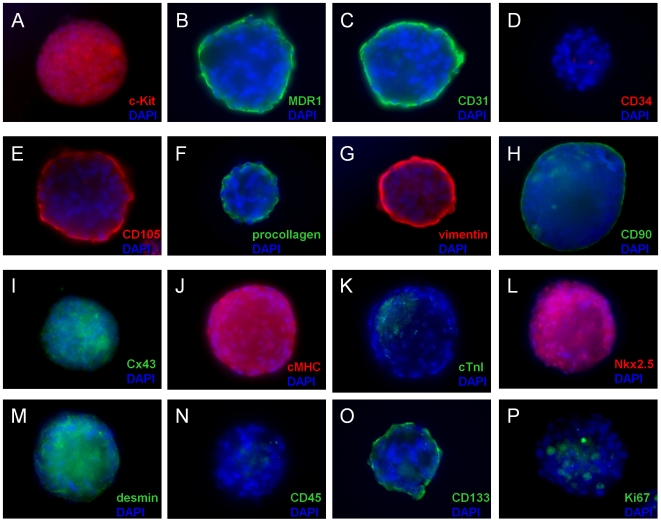
Immunophenotyping of cardiospheres. Widefield images of representative cardiospheres immunostained for 16 different cardiac-related antigens.

As shown above, the direct outgrowth from cardiac samples contains a mixed cell population of CPCs (c-Kit^+^), endothelial cells (CD31^+^, CD34^+^) and mesenchymal cells (CD90^+^). To explore the effects of three dimensional sphere culture on these sub-populations, we examined human cardiospheres for expression of CPC-related proteins (c-Kit, MDR1), mesenchymal-related proteins (CD105, CD90, procollagen type I, vimentin), endothelial-related proteins (CD105, CD133, CD31, CD34), hematopoietic-related proteins (CD34, CD45), cardiomyocyte-related proteins (Cx43, cMHC, cTnI, Nkx2.5 and desmin), and Ki67 to identify proliferative cells. As shown in Figure 5, cardiospheres consist of distinct layers with expression of the CPC marker c-Kit in the core (Figure 5A) and the mesenchymal marker CD105 on their periphery (Figure 5D). During cardiosphere culture, proliferative cells (Ki67 positive) were found primarily in the core of the sphere (Figure 5P). Many cardiospheres manifest cardiomyocyte-specific sarcomeric proteins (αMHC, cTnI) in the peripheral cells, indicative of partial myogenic differentiation (Figure 5J and 5K). On their periphery, cardiospheres also expressed CD31 (Figure 5C), CD133 (Figure 5O) and MDR-1 (Figure 5B). In their core, cardiospheres also often expressed connexin 43 (Figure 5I), Nkx2.5 (Figure 5L), and desmin (Figure 5M). Only rarely was MDR-1 expressed in the core of human cardiospheres (∼5%, not shown). Cardiospheres were consistently negative for CD34 (Figure 5D) and CD45 (Figure 5N). We conclude that core cells have a cardiac progenitor immunophenotype dominated by the expression of stem cell and cardiomyocyte-related antigens. Peripheral cells represent spontaneous differentiation of precursor cells into endothelial, mesenchymal, or cardiomyogenic lineages, and/or the encapsulation of core progenitors by a subset of supportive cardiac mesenchymal cells. Thus, when CPCs are cultured directly from myocardial tissue by carefully-established methods, further sub-culture permits the formation of self-organizing cardiospheres that create a complex, niche-like environment favoring the proliferation of cardiac progenitors in their core and a surface phenotype marked by mesenchymal- and cardiac-specific antigens [19], [20].

### CDCs are clonogenic, multipotent and capable of self-renewal

**Figure 6:**
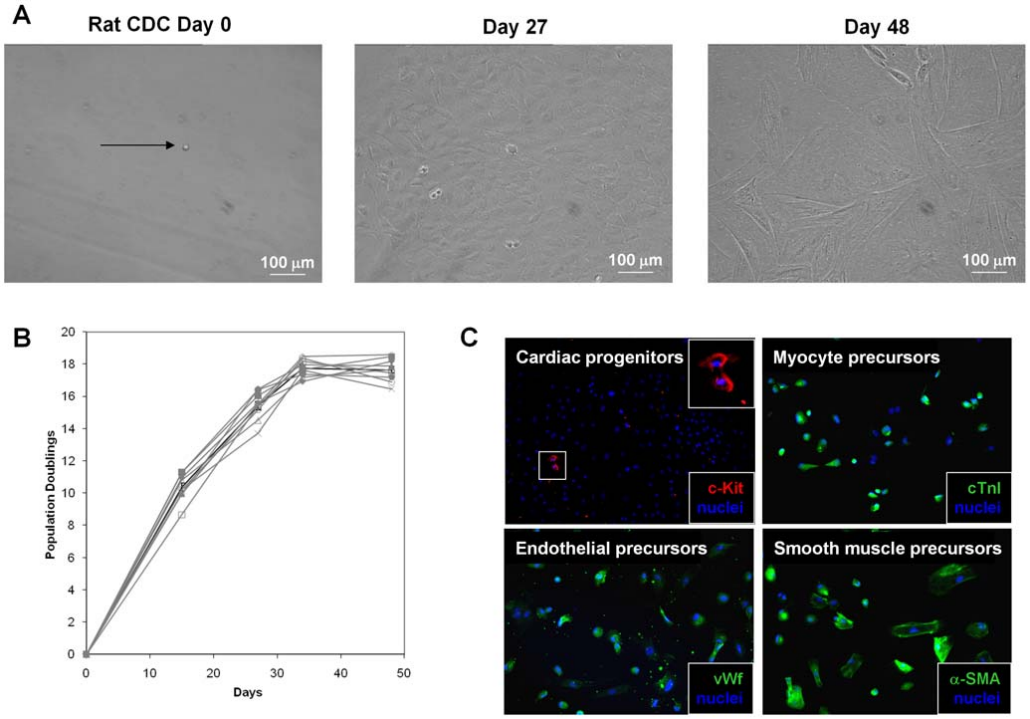
CDCs are self-renewing, clonogenic, and multipotent. (A) Phase image of a single plated rat CDC (Day 0) demonstrating proliferation (Day 27) and spontaneous morphological changes (Day 48). (B) Proliferation of nine clonal cell lines. (C) Differentiation of clonogenic cells into endothelial precursors (vWF), myocyte precursors(cTNI) and smooth muscle precursors (α-SMA). Also shown are clusters of cardiac progenitors (c-Kit) that persist during long-term culture.

CDCs, derived from rat specimens, readily formed clones when plated as single cells (Figure 6). Nine clonal cell lines were developed (from an initial 65 visually-verified single cells) which had an average doubling time of 42.2±0.7 hours during the first month. Clones proliferated for one month before proliferation spontaneously slowed and morphological changes occurred in most cells. After two months in culture (and 17.6±1.8 population doublings), clonal CDC populations exhibited spontaneous phenotypic heterogeneity. A small fraction of c-Kit expressing cells (4.9% by flow cytometry) could be seen dividing among still-proliferative groups of cells; other cells expressed cardiac troponin I (cTnI), α-smooth muscle actin (α-SMA), or von Willebrand factor (vWf), indicative of multipotentiality with a propensity to myocyte, smooth muscle, and endothelial lineages *in vitro*. These findings, as well as those with cardiosphere immunostaining (Figure 5 and related text above), are inconsistent with the sweeping claim that cardiosphere cells are fibroblasts lacking cardiomyogenic or endothelial differentiation potential [14].

## Discussion

This study demonstrates the broad applicability of techniques used to culture and expand CPCs from myocardial tissue. We have shown that CPCs can be grown from human, pig, mouse and rat tissue with very little identity deviation in CPC, endothelial and mesenchymal subpopulations. These data provide strong support for the concept that endogenous CPCs can be successfully grown for autologous cellular cardiomyoplasty [5], [8], [10]–[12], [15]. The clinical utility of CDC technology remains conjectural, but a phase 1 clinical trial with intra-coronary administration of autologous CDCs has begun, with evaluation of safety as the initial goal (CADUCEUS; see ClinicalTrials.gov).

Recent studies have confirmed our observation that technical deviance transforms the culture phenotype [13]. Shenje et al. demonstrated that culture with altered media following retrograde perfusion inconsistently provides outgrowth. These investigators attribute this finding to the purging of retained hematological cells responsible for generating outgrowth, as their limited series (n = 4) clustered culture failures in the retrograde perfusion group. Our series of successful cultures with retrograde perfusion (n = 45) in the absence of hematological cells (CD45^−^, lin^−^) disproves the notion that blood-derived elements figure prominently in the generation of CPCs from cardiac biopsies. The findings of Shenje et al. may reflect non-random clustering, as C57 WT mice inconsistently proliferate outgrowth, and retrograde perfusion has negligible effects on the generation of outgrowth from myocardial samples.

In a separate study, Andersen and colleagues grew cardiospheres from neonatal WK rats using unconventional methods [14]. Although critical of the literature, Andersen et al. did not attempt to reproduce published methods for the preparation of cardiospheres. Instead, the authors used a novel source tissue (neonatal rat heart); they stored the tissue in an untested solution; they added a filtering step which is not part of published protocols; and, they used harsh enzymatic detachment to collect cells from explant culture (5 min of trypsin treatment). These investigators found that neonatal WK rat cardiospheres contain a significant population of hematopoietic progenitors (as indicated by CD45). Of note, Andersen et al. 's non-hematological cells (CD45-) consist overwhelmingly of cardiac fibroblasts, as evidenced by the presence of collagen I. This result is likely due to one or more of the technical variations which they introduced. Andersen et al. further attributed the sparse cardiomyogenic activity they observed to contamination by myocardial fragments that could be removed by filtering the outgrowth. Our studies using myocardial lineage tracing debunk the importance of the postulated myocyte contamination, as we demonstrate that the presence of cardiomyocytes in the cellular outgrowth is not required for the generation of CPCs. We do not question the possibility that product filtration after prolonged enzymatic treatment may enrich in fibroblasts while selecting against genuine CPCs. Andersen et al. did not use human tissue, nor did they attempt to reproduce published successful protocols for isolating cardiac stem cells from animal or human tissue. Accordingly, their work provides no justification for the indictment of the clinical utility of cardiospheres.

In conclusion, we have demonstrated that, using established methods, expansion and proliferation of CPCs is simple and straightforward. This study has also shown that subtle variations in technique markedly alter phenotype and severely limit comparisons that can be made using variant methods. Finally, genetic lineage tracing has demonstrated that a portion of CPCs arise from myocardial dedifferentiation with the remainder originating proliferation of endogenous progenitors.
